# Dynamic minimum set problem for reserve design: Heuristic solutions for large problems

**DOI:** 10.1371/journal.pone.0193093

**Published:** 2018-03-15

**Authors:** Mathieu Bonneau, Régis Sabbadin, Fred A. Johnson, Bradley Stith

**Affiliations:** 1 Department of Wildlife Ecology and Conservation, University of Florida, 110 Newins-Ziegler Hall, P.O Box 110430, Gainesville, Florida, 32611-0430, United States of America; 2 URZ UR143, INRA, 97170, Petit-Bourg (Guadeloupe), France; 3 Applied Mathematics and Computer Science Unit, INRA-UR875, 24 Chemin de Borde Rouge, Auzeville, CS 5267, 31326 Castanet Tolosan Cedex, France; 4 Wetland and Aquatic Research Center, U.S. Geological Survey, 7920 NW 71 Street, Gainesville, Florida, 32653, United States of America; University of Waikato, NEW ZEALAND

## Abstract

Conversion of wild habitats to human dominated landscape is a major cause of biodiversity loss. An approach to mitigate the impact of habitat loss consists of designating reserves where habitat is preserved and managed. Determining the most valuable areas to preserve in a landscape is called the reserve design problem. There exists several possible formulations of the reserve design problem, depending on the objectives and the constraints. In this article, we considered the dynamic problem of designing a reserve that contains a desired area of several key habitats. The dynamic case implies that the reserve cannot be designed in one time step, due to budget constraints, and that habitats can be lost before they are reserved, due for example to climate change or human development. We proposed two heuristics strategies that can be used to select sites to reserve each year for large reserve design problem. The first heuristic is a combination of the Marxan and site-ordering algorithms and the second heuristic is an augmented version of the common naive myopic heuristic. We evaluated the strategies on several simulated examples and showed that the augmented greedy heuristic is particularly interesting when some of the habitats to protect are particularly threatened and/or the compactness of the network is accounted for.

## Introduction

Today, one of the main causes of the loss of biodiversity is the conversion of wild habitats to human dominated landscapes, for example for agricultural uses, oil and gas exploitation or urbanization [1, 2]. Conversion of land generally results in habitat destruction or degradation and habitat fragmentation, which dramatically changes the functioning of an ecosystem, i.e. its ability to provide food, water, cover and space to the native species and humans [3]. As a result, the remaining land may be inadequate to prevent the extinction of native species. One approach to mitigate negative human effects consists of designating reserves [4] where habitat is preserved and possibly managed. Determining the most valuable areas to preserve in order to support human development while ensuring species/population viability is called the *reserve design problem*.

Different formulations of the reserve design problem are available, depending on the objectives such as whether the reserve can be implemented immediately or over multiple years [5, 6]. This difference is known as the *static reserve design problem* (SRDP) versus the *dynamic reserve design problem* (DRDP). In the former, adapted, for example, to the construction of marine protected area [7, 8], the reserved sites are determined once as if the sites will all be purchased simultaneously [9, 10]. In the latter, the reserve cannot be implemented all at once, but sites have to be added to the reserve in an iterative way, over multiple years [11–14]. This may be the case, for example, if the necessary budget to create the reserve is not available instantaneously but is apportioned annually.

Common objectives of both approaches are to maximize representation, i.e. the number of species present in the reserve network at the end of the planning period [12, 15] or to maximize retention, i.e. both the number of species present inside and outside the reserve at the end of the planning period [14]. Another common objective consists of minimizing the cost of the reserved network while meeting a set of biodiversity targets [16–20]. This problem has been formulated using different frameworks and has various names in the literature, such as *minimum set problem*, *set covering problem*, *minimum area problem*, or *minimum representation problem*. In the rest of the article, we will simply use DRDP for reference to the problem of minimizing the cost of the reserved network while meeting a set of biodiversity targets. In the static case, the Marxan software [21] is a well-known software which uses *simulated annealing* to find a near-optimal reserve network. In the dynamic case, there does not exist universally recognized approaches yet. Although the Marxan solution can be used in a sequential way, i.e. the optimal reserve for the static problem is computed and then sites are acquired progressively according to the yearly available budget, this approach is not ideal. In the dynamic case, the future state of the landscape is uncertain and as a consequence, the optimal static solution can exhibit poor efficiency if implemented dynamically [22]. The major drawback of this approach is that sites can be lost before they can be purchased due for example to urban development. A natural way of framing the DRDP is to use the Markov Decision Process (MDP) framework. Then, MDP solution methods can be used, e.g. Stochastic Dynamic Programming (SDP) [4, 15, 23], allowing computation of the optimal reserve policy that accounts for several sources of uncertainty, such as the yearly budget, environmental losses and urban development or future site prices [12, 14, 15, 24]. The output of the SDP approach is an optimal reserve policy, or in other words, a function that returns the next sites to reserve for each possible state of the landscape (i.e. sites availability, biodiversity features and sites costs) and the yearly budget. Each year, the decision made by the optimal reserve policy is optimal in expectation over all possible future landscape availability changes and future budgets. But unfortunately, as in other applications, SDP can be used only for relatively small size problems, e.g. 10 sites with a 6 years planning period and a fixed yearly budget that allows to reserve only one site per year in [15]. In this article we are interested in large DRDP and we describe and explore approximate solution methods.

A common SDP alternative is to base the decision on heuristic policies, which either ignore the uncertainty on the future landscape, e.g., the *naive myopic* [15], or account for uncertainty only one time step ahead, e.g., the *informed myopic* [15]. Improvement of the naive myopic strategies has been proposed in [24] with the so-called site-ordering algorithm. It is based on the idea that the set of sites solution of the static problem should be computed first, and then the optimal purchasing order of the sequence should be determined subsequently, based on the expected value of a sequence.

In this article we first propose an extension of the site-ordering algorithm to solve the DRDP, by pairing the Marxan algorithm with the mechanism of the site-ordering algorithm. Second, we propose a generalization of the common naive myopic heuristic, particularly relevant when compactness of the network is important and rare biodiversity features can be lost during the construction of the reserve network.

We start by framing the DRDP as a MDP, which allows all the tested reserve policies to be described in a unifying framework.

## Material and method

### Problem description

To prevent future land conversion (e.g. urban development or agricultural use) and to be able to support native wildlife in the long term, we assume that *J* key habitats have been identified. These habitats are critical for the native species and we assume that target areas *H*_1_, …, *H*_*J*_ of each type of habitat should be reserved to help their conservation. *H*_1_, …, *H*_*J*_ could be seen as the minimum areas of target habitats that should be present in the landscape in order to support native wildlife.

Assume that there is a set *S*^*t*^ of available sites at time *t*. Each site *s* ∈ *S*^*t*^ can be potentially included into the network of reserved sites in order to help satisfy the habitat targets. Sites are characterized by their value to each habitat, defined by the amount of each type of habitat in the site *h*(*s*) = (*h*_1_(*s*), …, *h*_*J*_(*s*)). The cost in $ of purchasing a site is *c*(*s*). The set of sites that have been reserved during time period *t* is denoted *N*^*t*^; and ${\bar{N}}^{t}=\cup_{t^{\prime }=1}^{t-1}N^{t^{\prime }}$ is the set of reserved sites at the beginning of time period *t*. Thus, ${\bar{N}}^{t+1}={\bar{N}}^{t}\cup N^{t}$ is the network of reserved sites at the end of time period *t* or, equivalently, at the beginning of time period *t* + 1. Finally, ${\bar{N}}^{0}$ is the set of sites that are already in the reserve network at the beginning of the decision problem.

Here we study the problem of choosing a set of sites achieving a minimal representation of target habitats, as we were inspired from the real-world problem of extension of the Everglades Headwaters National Wildlife Refuge (EHNWR) [25]. But the framework that we propose can be used for any minimum representation problem. For example, *J* can be the number of species that are concerned by a conservation program and for all sites *s*, *h*_*j*_(*s*) equals 1 if species *j* is present in site *s* and 0 otherwise. *H*_1_, …, *H*_*J*_ are then the minimum number of reserved sites where the species should be present. We use this set-up and compare some of our proposed heuristics to the problem described in [15]. Results are discussed in S3 File.

A site *s* ∈ *S*^*t*^ not reserved at time *t* can be *converted* during the next time step with a conversion probability *μ*(*s*). This is the case for example when the site is bought and developed. The set of converted sites at the end of time period *t* is *L*^*t*^. If *s* is either converted or reserved, it is removed from the set of available sites. Thus, we have

$$\begin{array}{c} S^{t+1}=S^{t}\setminus (L^{t}\cup N^{t}), \end{array}$$ (1)

where . \ . is the set subtraction operator. For example the subtraction of the set of sites {1, 2} to the set {1, 2, 3, 4} is {3, 4}, i.e. {1, 2, 3, 4} \ {1, 2} = {3, 4}.

Not all sites can be reserved in one year and the set *N*^*t*^ has to satisfy the available budget *B*^*t*^. The set of sites *N*^*t*^ which are reserved at time *t* is *feasible* when the cost of reserving all sites in *N*^*t*^, i.e. ∑_*s*∈*N*^*t*^_ *c*(*s*), does not exceed *B*^*t*^, the available budget for reservation at time period *t*:

$$\begin{array}{ccc} N^{t}\;\text{is}\;\text{feasible}\; & \Leftrightarrow & \sum_{s\in N^{t}}c(s)\leq B^{t}. \end{array}$$

A *reserve design policy* is a decision rule describing for each year the feasible set of sites to reserve. A simple example is the *cheapest sites first* policy, where each year the cheapest sites are purchased until the budget is consumed. Mathematically, a reserve design policy is a function $\delta :\left(S^{t},{\bar{N}}^{t}\right)\to N^{t}$ that decides the feasible set *N*^*t*^ ⊆ *S*^*t*^ to reserve as a function of the currently available and already reserved sites, $\left(S^{t},{\bar{N}}^{t}\right)$. Note that, if all sites have a positive conversion probability (*μ*(*s*) > 0), then, whatever reserve design policy is chosen, as *t* increases, *S*^*t*^ will become empty with probability 1. Let $\bar{N}$ be the set of reserved sites as *t* → +∞.

The objective of the DRDP is to find a reserve design policy which selects with high probability (the only source of uncertainty being site development) a fixed-point reserve network $\bar{N}$ achieving all habitat targets:

$$\begin{array}{c} \forall j=1\ldots J\;\sum_{s\in \bar{N}}h_{j}(s)\geq H_{j}, \end{array}$$

at a minimal cost.

Finally, the compactness of the the network can also be important. Let us define the *extended cost* of a reserve network $\bar{N}$:

$$\begin{array}{c} c(\bar{N})=\sum_{s\in \bar{N}}c\left(s\right)+Penalty\left(\bar{N}\right)+\text{BLM}*\text{Boundary}(\bar{N}), \end{array}$$ (2)

The extended cost is used to evaluate the final reserve network $\bar{N}$ and should be minimized. Boundary quantifies the compactness of $\bar{N}$, as the length of the boundary surrounding the reserve network:

$$\begin{array}{c} \text{Boundary}(\bar{N})=\sum_{s\in \bar{N}}\pi_{s}-2\sum_{(s_{1},s_{2})\in \bar{N}}\pi_{s_{1},s_{2}}. \end{array}$$ (3)

*π*_*s*_ is the boundary length of site *s*, *π*_*s*_1_,*s*_2__ is the length of the shared boundaries between sites *s*_1_ and *s*_2_, and $(s_{1},s_{2})\in \bar{N}$ represents all the pairs of distinct sites in the reserve network. Fragmented networks will have greater boundary length value. BLM is a constant used to scale the influence of the Boundary values relative to cost and penalty factors, thereby influencing the compactness of the reserve network [21].

$Penalty(\bar{N})$ penalizes reserve networks which cannot meet all habitat targets. $Penalty(\bar{N})$ is equal to zero if the network $\bar{N}$ can meet all the habitat targets and is equal to a constant *α*, arbitrarily large but finite when at least one habitat target is not met. In this article, *α* is arbitrarily fixed to twice the cost of the initial landscape: *α* = 2 * ∑_*s*∈*S*^0^_ *c*(*s*).

We can now define the *expected extended cost* of a feasible policy *δ* in a reserve design problem with initial set of available sites *S*^0^ and initial reserve network ${\bar{N}}^{0}$, as:

$$\begin{array}{c} EEC_{\delta }(S^{0},{\bar{N}}^{0})=\sum_{{\bar{N}}^{0}\subseteq \bar{N}\subseteq \left(S^{0}\cup {\bar{N}}^{0}\right)}P_{\mu }(\bar{N}|S^{0},{\bar{N}}^{0},\delta )\times c\left(\bar{N}\right). \end{array}$$ (4)

Intuitively, *EEC*_*δ*_ is the value of the extended cost that one can expect if the reserve design policy *δ* is used. Here the summation is over all fixed-point reserve networks $\bar{N}$ that can be reached when the reserve design policy *δ* is used and when the initial state is $\left(S^{0}\cup {\bar{N}}^{0}\right)$. In this summation, it is necessary to consider every possible scenario of sites conversion. For example, consider a problem with three sites (1, 2, 3) with unitary cost, where *B*^*t*^ = 1 for any time step *t*, and where any combination of two sites can reach habitat targets. Suppose that our policy *δ* reserves the first site during the first time step, i.e. *N*^0^ = {1}. Then, site 2 is reserved during the second time step if it is available. Finally, *δ* reserves site 3 if site 2 is not available or reserves no site when all are converted. Note that the value of *N*^0^ is always deterministic and only depends on the policy. Because only one site is reserved during the first time step, the two other sites can be converted or not at the end of the first time step. There are thus 4 possible values of *S*^1^: a first one where no sites are converted (then *N*^1^ = {2}), two scenarios where only one site is converted (then *N*^1^ = {2} or *N*^1^ = {3}) and a final scenario where all sites are converted *N*^1^ = ∅. Each of these scenarios results in a fixed-point reserve network, which has to be included in the summation. Then, $P_{\mu }(\bar{N}|S^{0},{\bar{N}}^{0},\delta )$ is the probability that the fixed-point reserve network $\bar{N}$ will be observed, or equivalently the probability of the associated site conversion scenario.

Finally, noting Δ the set of feasible reserve design policies for a given DRDP, the *optimal reserve design policy* δ* (for a given pair of sets of initial available and developed sites) is defined as:

$$\begin{array}{c} \delta^{*}=\text{arg}\underset{\delta \in \Delta }{\text{min}}EEC_{\delta }(S^{0},{\bar{N}}^{0}). \end{array}$$ (5)

Thus, the optimal reserve design policy is the one with minimal expected extended cost. In the following section, we show that the problem of finding an optimal reserve selection policy can be modelled as a stationary, infinite-horizon *Markov Decision Process* [26]. The MDP framework offers several exact (or approximate when the problem is too large to solve exactly, as is the case here) solution algorithms and allows presenting various heuristic methods in a unifying framework. The DRDP problem has already been modelled as a MDP, in [15] and [27]. However, in the first article, only very small problems are dealt with, considering a very short time horizon. The second article deals with large stationary, infinite-horizon problems, for which approximate solution methods are proposed. However it assumes that only one site can be reserved at each time step, which limits its applicability.

### Framing the DRDP as a Markov Decision Process

#### Markov Decision Processes

A *stationnary, infinite horizon Markov Decision Process (MDP)* is defined by a 4-tuple <X,A,P,r>, where:

- X is the finite set of possible states of the world.
- A is the finite set of allowed actions.
- P:X×A×X→[0,1] is a state transition probability. $P(x^{\prime }|x,a)$ is the probability that if the current state of the world is *x* and the applied action is *a*, then the following state is *x*′.
- r:X×A→ℜ is an instant reward function. *r*(*x*, *a*) is the instant reward obtained when action *a* is applied in state *x*.

In a stationary infinite horizon MDP, a stationary policy δ:X→A assigns an action at any time step. *Solving* a MDP amounts to finding an *optimal* policy. The *value function* $V_{\delta }:X\to ℜ$, associated with an arbitrary policy *δ* is defined as:

$$\begin{array}{c} V_{\delta }\left(x\right)=E[\sum_{t=0}^{+\infty }r(X^{t},\delta \left(X^{t}\right))|X^{0}=x]. \end{array}$$ (6)

Note that the expectation of the infinite sum may not be finite in the general case. Therefore, in general, one considers *discounted* infinite-horizon MDPs, instead of undiscounted ones. That is, the reward at time *t* is discounted by a factor *γ*^*t*^, where 0 < *γ* < 1. This guarantees that the expected value remains finite. However, in the DRDP case, with probability 1 an absorbing state with associated reward 0 is reached in a finite number of steps. This guarantees that *V*_*δ*_(*x*) is finite, even in the undiscounted case.

An optimal policy is then a policy which maximizes *V*_*δ*_ in every starting states:

**Definition 1** (**MDP optimal policy**) *Let* <X,A,P,r> *be an infinite-horizon stationnary MDP*. $\delta^{*}:X\to A$ *is an optimal policy for the MDP if and only if it satisfies*:

$$\begin{array}{c} V_{\delta^{*}}\left(x\right)\geq V_{\delta }\left(x\right),\forall x\in X,\;for\;any\;policy\;\delta . \end{array}$$

It is a well-known fact that whenever *V*_*δ*_ takes finite values for all policies and all states, then a MDP admits an optimal policy (Puterman, 1994). Furthermore, such an optimal policy (and its value function) can be computed in a time polynomial in the number of elements of X and A, using *Dynamic Programming algorithms*, such as the *Value Iteration* or *Policy Iteration* algorithms (Puterman, 1994).

#### A Markov Decision Process model for DRDP

From the definition of the DRDP we have given, this problem seems to fit quite easily in the MDP framework. Indeed, let us define a MDP <X,A,P,r>, which optimal policies correspond to optimal policies of a given DRDP problem.

Let us consider a DRDP with *n* sites. Then, the state of the world at any time is uniquely defined by the knowledge of the pair $\left(S,\bar{N}\right)$ of available sites and current reserve network, where: $S,\bar{N}\subseteq \left\{1,\ldots ,n\right\}$ and $S\cap \bar{N}=\emptyset$.

Thus, for the corresponding MDP,

$$\begin{array}{c} X=\{x=\left(S,\bar{N}\right)|\left(S,\bar{N}\right)\subseteq {\left\{1,\ldots ,n\right\}}^{2}\;\text{and}\;S\cap \bar{N}=\emptyset \}. \end{array}$$ (7)

Note that the set *L* of developed sites can be deduced from *S* and $\bar{N}$: $L=\left\{1,\ldots ,n\right\}\setminus (S\cup \bar{N})$.

In a DRDP, actions correspond to feasible subsets of available sites to reserve. Note that the feasible set of actions of the DRDP, that is the feasible set of actions of the corresponding MDP, depends on the currently available sites, i.e. on the current MDP state *x*. Even though the MDP framework allows to define state-dependent actions sets, we find it more convenient to define an action set which is independent of the current state (but depends on time to account for budget variation in time) and to use the reward function to *forbid* some actions in some states. Thus, for the corresponding MDP we simply define:

$$\begin{array}{c} A^{t}=\{N\subseteq \left\{1,\ldots ,n\right\},\sum_{s\in N}c\left(s\right)\leq B_{t}\}. \end{array}$$ (8)

This means that we consider that, a priori, the reserve designer can choose any subset of sites to reserve at any time step, even when some sites might have already been converted (provided that the total reservation cost does not exceed the available budget).

We have to define the transition function $P(x^{\prime }|x,a)$ of the MDP corresponding to the DRDP. First, note that any x∈X corresponds to a pair $\left(S,\bar{N}\right)$ with $\left(S,\bar{N}\right)\subseteq {\left\{1,\ldots ,n\right\}}^{2}$ and $S\cap \bar{N}=\emptyset$ in the DRDP. In the same way, any action a∈A corresponds to a subset ∅ ⊆ *N* ⊆ {1, …, *n*}.

To define the transition function P, we will distinguish the case where *a* is unfeasible for *x* from the case where it is feasible:

- If *S* is non-empty, *N* ⊆ *S* and *N* is feasible in terms of cost, then, the set of possible successor states $\left\{x^{\prime }=\left(S^{\prime },{\bar{N}}^{\prime }\right)\right\}$ is defined by: (i) ${\bar{N}}^{\prime }=\bar{N}\cup N$ and (ii) *S*′ ⊆ *S* \ *N* and
  $$\begin{array}{c} P\left(x^{\prime }|x,a\right)=(\prod_{s\in S\setminus (N\cup S^{\prime })}\mu \left(s\right))\cdot (\prod_{s\in S^{\prime }}\left(1-\mu \left(s\right)\right)). \end{array}$$ (9)
- In the case where *S* is empty, the only feasible action is *a* = *N* = ∅ and we simply have $P(x^{\prime }=x|x,a)=1$.
- In all other cases, i.e. *a* unfeasible, we let $P(x^{\prime }=x|x,a)=1$ as well.

We define a reward function $r\left(x^{t},a^{t}\right)=r(\left(S^{t},{\bar{N}}^{t}\right),N^{t})$, such that:

- If *S*^*t*^ ≠ ∅, i.e. some sites are still available:
  - If *N*^*t*^ is feasible, i.e. *N*^*t*^ ⊆ *S*^*t*^ and ∑_*s*∈*N*^*t*^_ *c*(*s*) ≤ *B*^*t*^ and *S*^*t*+1^ ≠ ∅, an instant reservation cost is incurred:
    $$\begin{array}{c} r\left(x^{t},a^{t}\right)=-\sum_{s\in N^{t}}c\left(s\right), \end{array}$$
  - If *N*^*t*^ is feasible, i.e. *N*^*t*^ ⊆ *S*^*t*^ and ∑_*s*∈*N*^*t*^_ *c*(*s*) ≤ *B*^*t*^ and *S*^*t*+1^ = ∅, then the reserve design problem is over. An instant reservation cost is incurred, as well as penalties linked to the conservation targets and to the reserve boundary length:
    $$\begin{array}{c} r\left(x^{t},a^{t}\right)=-\text{BLM}*\text{Boundary}({\bar{N}}^{t}\cup N^{t})-Penalty\left({\bar{N}}^{t}\cup N^{t}\right). \end{array}$$
  - If *N*^*t*^ is not feasible, *r*(*x*^*t*^, *a*^*t*^) = −∞.
- If *S*^*t*^ = ∅, i.e. all sites are either converted or reserved,
  - If *N*^*t*^ = ∅, then *r*(*x*^*t*^, *a*^*t*^) = 0.
  - If *N*^*t*^ ≠ ∅, then *r*(*x*^*t*^, *a*^*t*^) = −∞.

Note that *r*(*x*^*t*^, *a*^*t*^) = −∞ is used to prohibit the cases where the action *a*^*t*^ is willing to reserve sites that are not available. In practice, we assign *r*(*x*^*t*^, *a*^*t*^) an arbitrary large negative value.

One can easily check that any reservation policy can be modeled as an MDP policy in the corresponding MDP. Furthermore, it is also possible to show that a reservation policy has finite value in all states if and only if it chooses only feasible sets of sites to reserve, in all configurations of sites. Finally, we can check that:

$$\begin{array}{c} V_{\delta }\left(S^{0},{\bar{N}}^{0}\right)=EEC_{\delta }\left(S^{0},{\bar{N}}^{0}\right). \end{array}$$

Thus, we have modeled the Dynamic Reserve Design Problem as one of solving a stationary infinite horizon MDP. We will see, in the following section, that even though we face a classical MDP, usual MDP solution algorithms (*value iteration*, *policy iteration*, *linear programming*…) are not able to solve this problem, due to the huge size of its state (and action) spaces. Some Artificial Intelligence methods, based on simulation, may be able to solve problems with more sites, however are still limited. Therefore, we propose heuristic approaches, extending the myopic approach and the well-known Marxan and myopic heuristics to the dynamic framework, to solve this problem approximately.

### Solution method

#### Exact method

A common approach that can be used for small problem, e.g. only 10 sites in [15] or 12 sites in [22], consists in using a *backward induction algorithm*. The *backward induction algorithm* is based on a particular organization of the computation: starting from the end. First, one can see that the value of a policy can be computed recursively. Indeed, it is well known (see, e.g. [26]) that Eq (6) is equivalent to:

$$\begin{array}{ccc} V_{\delta^{*}}(x) & = & \underset{a}{\text{max}}\sum_{x^{\prime }}P(x^{\prime }|x,a)(r(x,a)+V_{\delta^{*}}(x^{\prime })),\\ \delta^{*}(x) & = & \text{arg}\underset{a}{\text{max}}\sum_{x^{\prime }}P(x^{\prime }|x,a)(r(x,a)+V_{\delta^{*}}(x^{\prime })). \end{array}$$ (10)

Second, let us define $F=\{x=\left(S=\emptyset ,\bar{N}\right)\}$, the set of final states. Note that, if *x* ∈ *F*, we have:

- *p*(*x*′ = *x*|*x*, *a* = ∅) = 1 and *r*(*x*, *a* = ∅) = 0, for the only applicable action, *a* = ∅, and
- *p*(*x*′ = *x*|*x*, *a*) = 1 and *r*(*x*, *a*) = −∞, for any non-applicable action, *a* ≠ ∅.

Thus, obviously, δ*(*x*) = ∅, ∀*x* ∈ *F* and *V*_δ*_(*x*) = 0, ∀*x* ∈ *F*, *V*_δ*_(*x*) = −∞, ∀*x* ∉ *F*.

Then, we can pursue a *backwards induction* approach. We consider states $x=(S,\bar{N})$ in increasing size of *S*:

- Any state $x=(S,\bar{N})$ with |*S*| = 1 and *a* ≠ ∅ can only transition to states $x^{\prime }=\left(S^{\prime }=\emptyset ,{\bar{N}}^{\prime }\right)$. Thus, *V*_δ*_(*x*′) has already been computed for all such *x*′ and δ*(*x*) can be computed using Eq (10).
- Then, we can progressively compute δ*(*x*) for states $x=(S,\bar{N})$, by increasing size |*S*| = 2, |*S*| = 3… to obtain the optimal policy.

#### Reinforcement-learning and AI methods

Applying an exact dynamic programming approach requires computing δ*(*x*) and *V*_δ*(*x*)_ in turn for all potential states $x=(S,\bar{N})$ with $S\cap \bar{N}=\emptyset$. The number of computations is huge (>> 2^*n*^). In addition, the number of potential successors of $x=(S,\bar{N})$ is also huge (2^*n*^), therefore, a single computation can be very costly to compute. This means that exact dynamic programming is currently restricted to solving very small problems. *Reinforcement Learning* approaches [28] use simulations of the MDP dynamics and sampling to approximate δ*. For example, [27] have proposed to use a linear approximation of *V*_δ*(*x*)_, together with a sample-based approach to compute approximate policies.

This approach is promising, however it has several drawbacks:

1. It provides no guarantee about the suboptimality of the approximate policy,
2. It only considers unit budget limitations (one site can be reserved at each time step) and,
3. it is not well adapted to global constraints such as Boundary costs.

In this article, we propose heuristic approaches, based on a dynamic version of Marxan and several augmented heuristics, to compute approximate policies. Even though no performance guarantees are available for this method, it can easily consider global constraints and non-unit costs and is quite fast and robust (see Results section).

#### A dynamic version of Marxan

Marxan [21, 29, 30] was initially designed to solve the SRDP. In this work, we propose a dynamic version of Marxan, where at each decision step, the reserve network is computed using Marxan as usual, sites are then sorted by increasing priority, purchasing higher priority sites first, until the yearly budget is exhausted.

We first show that the Marxan solution can be viewed as a solution of a particular set-up of our MDP: (i) with an infinite budget at the first time step and (ii) by defining the transition probability such that all non-reserved sites are getting developed after the first time step. Here the reward function to maximize is defined as follows:

$$\begin{array}{c} r_{Marxan}(N)=-\sum_{s\in N}c(s)-\text{BLM}*\text{Boundary}(N)-\sum_{j=1}^{J}{\text{SPF}}_{j}*\text{Penalty}(j)-\text{CostThreshold}(N). \end{array}$$ (11)

Penalty is used to ensure that the habitat targets are met for every habitat types. If the network *N* does not meet the target for a specific habitat type *j*, then Penalty(*j*) represents an additional minimal cost needed to reach the target for this habitat. Again, SPF is used to define some sort of habitat priority. Finally, a CostThreshold function is used in case a maximal budget is available. The function is set extremely high when the cost of the network *N* is higher than the budget and to zero otherwise. As we are considering an unlimited budget, we fixed CostThreshold to zero. In addition, we will not use habitat priority, considering that all habitats are of equal ecological interest. Nonetheless, we use SPF = 1×10^6^ to ensure that the computed network meets the target for every habitats. Indeed, using a low SPF value can lead to networks that are not necessarily meeting exactly the target when it allows a large cost saving.

The Marxan software uses a simulated annealing approach to find a near-optimal reserve design ${\bar{N}}_{Marxan}^{*}$ maximizing *r*_*Marxan*_. More information on the reward function and the use of this method is available in the Marxan manual [30] or in [29]. In all our experiments, Marxan solutions ${\bar{N}}_{Marxan}^{*}$ were computed using R and the R-package described in [31].

In practice, ${\bar{N}}_{Marxan}^{*}$ represents a reserve network of minimal cost that meets the habitat targets, but a major issue is that this network cannot be implemented within a single year, due to budget limitation. Then, some sites can be converted before being purchased. The purchasing order of the sequence ${\bar{N}}_{Marxan}^{*}$ is of first importance to minimize the risk for sites in ${\bar{N}}_{Marxan}^{*}$ to get developed before being reserved. This order should be based on the site conversion probabilities as well as the site values (i.e. costs and amounts of target habitats). To do this, we propose to adapt the work from [24] with the so-called site-ordering algorithm. Intuitively, a given purchasing order of a sequence is better than another one if it has a higher probability to meet every habitat targets. Let’s denote, *A*_*j*_(*N*) the abundance of habitat *j* in the reserve network *N*:

$$\begin{array}{c} A_{j}(N)=\sum_{s\in N}h_{j}(s). \end{array}$$

And *EA*_*j*_(*N*), the effective abundance of habitat *j* in the reserve network *j*:

$$\begin{array}{c} EA_{j}(N)=\text{min}(H_{j},A_{j}(N)). \end{array}$$

The effective abundance is the amount of reserved habitat, upper bounded by the amount that is required to be protected. A reserve network *N* that satisfies all the habitat target thus satisfies:

$$\begin{array}{c} \sum_{j=1}^{J}\frac{EA_{j}(N)}{H_{j}}=J. \end{array}$$

The problem in the dynamic case is that it can not be determined if ${\bar{N}}_{Marxan}^{*}$ will indeed verify the previous equation before all the sites have been purchased. A common practice in *decision theory* consists in taking a decision based on the expected value of the original criterion. Let *σ*_*Marxan*_ = {*s*_1_, …, *s*_*p*_} be a particular ordering of the sequence ${\bar{N}}_{Marxan}^{*}$. It is first necessary to estimate the purchasing year of any site in this sequence. It is necessary to use a fixed value of the yearly budget *m*_*B*_, which can be for example an average of the yearly budget one can expect to have over the planning period. Then it is easy to compute the purchasing year of a given site *s*_*i*_, denoted $t_{Buy}^{i}$:

$$\begin{array}{c} t_{Buy}^{i}=\left\lceil \frac{\sum_{i^{\prime }=1}^{i}c(s_{i^{\prime }})}{m_{B}}\right\rceil , \end{array}$$

Where ⌈.⌉ is the above rounding function. We can then define the expected value of the sequence’s abundance:

$$\begin{array}{c} E\left[A_{j}\left(\sigma_{Marxan}\right)\right]=\sum_{i=1}^{p}{\left(1-\mu \left(s_{i}\right)\right)}^{t_{Buy}^{i}-1}h_{j}\left(s_{i}\right). \end{array}$$

In this case, the abundance of a site is only accounted for if the site is not converted before it has been purchased, which happens with a probability ${\left(1-\mu \left(s_{i}\right)\right)}^{t_{Buy}^{i}-1}$. The expected value of the effective abundance is:

$$\begin{array}{c} E[EA_{j}(\sigma_{Marxan})]=\text{min}(H_{j},E[A_{j}(\sigma_{Marxan})]). \end{array}$$

Then, the optimal sequence $\sigma_{Marxan}^{*}$ is simply the sequence maximizing the value of the expected effective abundance:

$$\begin{array}{c} \sigma_{Marxan}^{*}=\text{arg}\underset{\sigma_{Marxan}}{\text{max}}(\sum_{j=1}^{J}E[EA_{j}(\sigma_{Marxan})]). \end{array}$$ (12)

Note that depending on the preferences of the decision maker, some other criteria could be used. The previous criterion can be considered as “utilitarian”, since reaching the habitat target for some species can compensate for others. An “egalitarian” decision maker, on his side, would prefer to minimize the risk that any habitat target be not met. The following definition of the optimal sequence could then be used:

$$\begin{array}{c} \sigma_{Marxan}^{*}=\text{arg}\underset{\sigma_{Marxan}}{\text{max}}(\underset{j}{\text{min}}(E[EA_{j}(\sigma_{Marxan})])). \end{array}$$ (13)

The dynamic version of the Marxan algorithm can be decomposed into the following steps:

1. Compute the optimal reserve network ${\bar{N}}_{Marxan}^{*}$ using the Marxan algorithm to solve the static problem.
2. Compute the optimal sequence $\sigma_{Marxan}^{*}$ and purchase the first sites of the sequence until the annual budget is spent.
3. Update the set of available sites (i.e. removed sites that are converted and sites that have been reserved) and go to 1., until all habitat targets have been met or there are no available sites in the landscape.

Note that when the remaining annual budget is lower than the cheapest site, it is automatically transferred to the next decision step. We used the same rule for all the tested strategies.

#### Greedy heuristics

Greedy, or naive myopic, heuristics consist in considering that only one site can be purchased per time period, while again considering that the problem is static. Each greedy heuristic is based on a reward function representing the value of a particular site. We will consider two different heuristics: the richness heuristic and the rarity heuristic. These heuristics are two variations of what is often called naive myopic heuristic and a complete description can be found in [30], pages 110–114. In the following, we propose a description using our MDP framework.

The richness heuristic consists in putting more value on sites that allow the highest gain in terms of effective abundance of habitats. The reward function can be defined as follows:

$$\begin{array}{c} r_{Rich}^{t}(s)=-\sum_{j=1}^{J}r_{Rich_{j}}^{t}(s). \end{array}$$ (14)

Where:

$$\begin{array}{c} r_{Rich_{j}}^{t}(s)=\{\begin{array}{cc} \frac{h_{j}(s)}{H_{j}*(c(s)+BLM*(Boundary({\bar{N}}^{t}\cup \{s\})-Boundary({\bar{N}}^{t})))} & \text{if}\;\sum_{s\in {\bar{N}}^{t}}h_{j}(s)<H_{j}.\\ 1 & \text{Otherwise}. \end{array} \end{array}$$

The richness heuristic tends to select sites with high ecological value first and as a consequence, habitats that are present in low quantity tend to be selected last. This is particularly problematic when sites can be converted over the year. It can lead to situations where some particular type of habitat, not frequent over the landscape, gets exhausted before the target is met. On the contrary, the rarity heuristic takes into account the initial amount of each habitat present in the landscape so as to select rare habitats first:

$$\begin{array}{c} r_{Rar}^{t}(s)=-\sum_{j=1}^{J}[\frac{\frac{EA_{j}[{\bar{N}}^{t}\cup \{s\}]}{\sum_{s^{\prime }\in S^{t}}h_{j}(s^{\prime })}}{c(s)+BLM*(Boundary({\bar{N}}^{t}\cup \{s\})-Boundary({\bar{N}}^{t}))}]. \end{array}$$ (15)

Using a greedy heuristic involves:

1. Purchasing the sites with highest rewards in an iterative fashion, until the yearly budget does not allow to purchase new sites anymore.
2. Update the set of available sites (i.e. removed sites that are converted and sites that have been reserved) and go back to 1.

#### Augmented greedy heuristics

Both previous heuristics do not account for the fact that some habitats can be more subject to conversion than others. We propose augmented greedy heuristics which attempt to weight each biodiversity feature automatically, allowing for example to account for the effect of habitat loss. We propose to use weights λ_1_, …, λ_*J*_ in the reward function of the two previous heuristics, such that some habitats are given more weight, for example to counter-balance a high conversion rate or the effect of habitat rarity:

$$\begin{array}{c} r_{ARich}^{t}(s)=-\sum_{j=1}^{J}\frac{\lambda_{j}}{\lambda_{c}}r_{Rich_{j}}^{t}(s). \end{array}$$ (16)

And

$$\begin{array}{c} r_{ARar}^{t}(s)=-\sum_{j=1}^{J}\frac{\lambda_{j}}{\lambda_{c}}[\frac{\frac{EA_{j}[{\bar{N}}^{t}\cup \{s\}]}{\sum_{s^{\prime }\in S^{t}}h_{j}(s^{\prime })}}{c(s)+BLM*(Boundary({\bar{N}}^{t}\cup \{s\})-Boundary({\bar{N}}^{t}))}]. \end{array}$$ (17)

Note that the weights for the augmented richness and augmented rarity are not necessarily equal and have to be computed separately. We define an extra weight λ_*c*_ that is used to model the contribution of the site’s extended cost.

Providing any set of weights λ^1^ and λ^2^ is sufficient to entirely define the reserve design policy $\delta_{ARich}^{\lambda^{1}}$ and $\delta_{ARar}^{\lambda^{2}}$. Then for the augmented richness and rarity heuristics, it is clear that the optimal set of weights are the weights resulting in the reserve policy with the highest expected extended cost. Finding the optimal set of weights requires solving one of the following optimization problems:

$${\left(\lambda_{1},\ldots ,\lambda_{J}\right)}_{ARich}^{*}=\text{arg}\underset{\left(\lambda_{1},\ldots ,\lambda_{J}\right)}{\text{max}}EEC_{\delta_{ARich}^{\lambda }}\left(S^{0},{\bar{N}}^{0}\right),$$ (18)

$${\left(\lambda_{1},\ldots ,\lambda_{J}\right)}_{ARar}^{*}=\text{arg}\underset{\left(\lambda_{1},\ldots ,\lambda_{J}\right)}{\text{max}}EEC_{\delta_{ARar}^{\lambda }}\left(S^{0},{\bar{N}}^{0}\right).$$ (19)

In order to solve (approximately) these optimization problems, we:

- Computed Monte-Carlo approximations $\hat{EEC_{\delta_{AR\cdot }^{\lambda }}}$, since the exact values are too costly to compute and
- Discretized the domains *D*_*j*_ of the variables λ_*j*_ and used a genetic algorithm provided by Matlab, to compute the optimal discrete values.

The genetic algorithm is simply searching for the effect of varying the value of the different weights and ultimately results in a near-optimal combination of these weights. For example, using a high value of λ_1_ compared to the other weights creates a reserve design policy that first selects sites with a large quantity of the first habitat. If using this rule of thumb is beneficial (i.e. translates to a high value of the expected extended cost), as is the case, for example, when the first habitat is subject to a high conversion rate, the genetic algorithm will save this combination and potentially return it, if no better combinations are found. What is particularly interesting is that the weights values are computed automatically, as a function of the landscape’s characteristics, such as relative quantity of each habitat and conversion rates.

To approximate the expected extended cost, we proposed a simulation approach, where the strategy is applied on simulated scenarios and the EEC is averaged. The simulation procedure is detailed in the section *Value of the reserve policies*.

### Experiments

We conducted simulation experiments to compare the dynamic Marxan heuristics, the two greedy heuristics and the two augmented greedy heuristics.

#### Comparison on a small landscape

**Fig 1:**
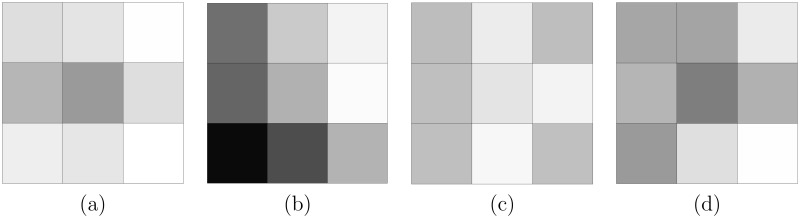
Landscape set-up in the small landscape example. There are 9 sites with unitary cost and unitary yearly budget. Amount of (a) the first target habitat *h*_1_ and (b) second target habitat *h*_2_. Conversion rate in the (c) non-correlated scenario and (d) correlated scenario.

We first compare the policies on a small simulated landscape in order to be able to compute the optimal policy using the *backward induction* algorithm. We simulate a landscape composed of 9 square sites of unit cost distributed on a grid of 3 rows and 3 columns. We consider two habitat targets, i.e. *J* = 2, and to determine the amount of habitat per site, we use a Gaussian random field. We use a third habitat which represents a non-target habitat, so that not all sites have target habitats. We use a Gaussian random field to be able to simulate spatially coherent habitats, which is a more realistic situation. We set the parameters of the model such that the first habitat is rare and the second habitat is more common. We use a Gaussian random field of mean equal to 5 for each habitat with the following scaling parameters of the co-variance structure: (5, 2) for the first habitat, (1, 2) for the second habitat and (1.5, 2) for the third habitat. The first parameter is the sill and the second parameter is the range of the covariance. We use an exponential covariance function and obtain a simulated landscape with 133,730m^2^ of *h*_1_ and 348,479m^2^ of *h*_2_. For both habitats, the targets *H*_1_ and *H*_2_ are fixed to 50% of the entire amount of this initial amount. The amount of each habitat in each site is presented in Fig 1a and 1b.

As in [24], we simulate conversion rates under two different scenarios: (i) a *non-correlated* scenario where the conversion rate and amount of target habitats are independent, and the conversion probability is drawn from a uniform distribution on [0.01; 0.3]; and (ii) a *correlated* scenario, where the conversion rate increases with the amount of target habitats in the site. More precisely, if a site has a proportion of *h*_1_ higher than 5%, its conversion rate is drawn from a uniform distribution on [0.02; 0.6] and from [0.001, 0.1] otherwise. Sites with a large proportion of the first target habitat are thus more threatened by conversion. The conversion probabilities are available in Fig 1c and 1d.

Finally, only one site per year can be purchased, i.e. *B*^*t*^ = 1 for all *t*. In addition, compactness is not accounted for and we use *BLM* = 0. In this case, only the cost of the network is minimized.

#### Comparison on large landscapes without compactness, i.e. BLM = 0

Except for the small landscape example, it is not possible to compute the exact expected extended cost of all the policies and simulation has to be used. As in [15], we propose to compute the expected extended cost of each strategy using a Monte Carlo approach. A scenario *τ* is a sampling of the possible land conversion when no reserve design policy is used and of the possible yearly budget:

$$\begin{array}{c} \tau =\{(S_{\tau }^{1},B_{\tau }^{1}),(S_{\tau }^{2},B_{\tau }^{2}),\ldots \} \end{array}$$

Each scenario ends when all sites are converted. Then we apply each policy separately on this scenario, where at each time step, the set of available sites is $S^{t}=S_{\tau }^{t}\setminus {\bar{N}}^{t}$ and the policy determines a feasible set of sites. The process ends when the targets are met for all habitats or when no sites are available. The EEC is finally computed accordingly. We use 1,000 simulated scenarios for each policy and approximated their EEC by averaging their results on the simulated scenarios.

We use the same set-up as in section **Comparison on a small landscape** but with 880 sites and introduce cost dissimilarity. We propose to base the site cost on the amounts of target habitats available in the site:

$$\begin{array}{c} c(s)=\$4,000*(h_{1}(s)+h_{2}(s)). \end{array}$$

**Fig 2:**
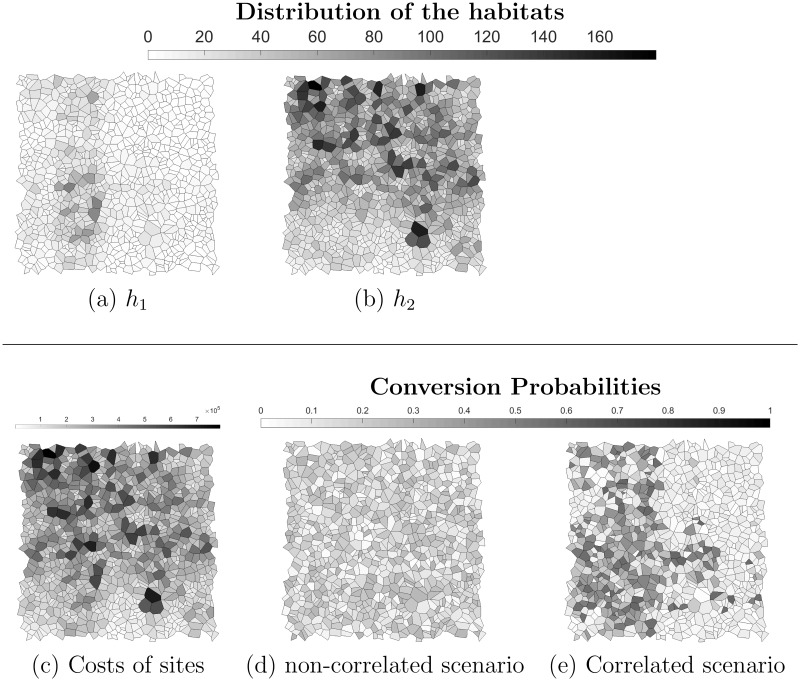
Landscape set-up in the large landscape without compactness. Amount of (a) the first habitat target *h*_1_ and (b) second habitat target *h*_2_. (c) Sites costs. Loss probability in the (d) non-correlated scenario and (e) correlated scenario.

The average price per site is $216,000 (range: $4,300–$790,000). We propose to simulate sites of random shape by first simulating the centroid of every sites using a uniform distribution and second determine site’s boundaries by computing a voronoï diagram out of the centroids using Matlab. The simulated landscapes, conversion probabilities and costs are available in Fig 2. The first habitat is rare in the landscape and is mostly found on a long strip located West. The second habitat is present on most sites but preferentially in the North part of the landscape as well as in the South East part. For this experiment, compactness is not accounted for and we use *BLM* = 0. We define a stochastic yearly budget equal to US$5,000,000 with probability $\frac{2}{3}$, US$3,000,000 with probability $\frac{1}{6}$ and US$1,000,000 with probability $\frac{1}{6}$.

#### Comparison on large landscapes with compactness, i.e. BLM > 0

We propose a last experiment where the compactness of the reserve network is also optimized using *BLM* > 0. The sites attributes and problem features are inspired from a real world problem, the extension of the Everglades Headwaters National Wildlife Refuge (EHNWR) and Conservation Area in central Florida. The extension of this reserve aims at contributing to the conservation of over 160 protected species living in the refuge boundaries and mitigate future effects of climate change and urbanization [25]. An extensive presentation of the problem parameters can be found online at https://globalchange.ncsu.edu/secsc/wp-content/uploads/014-Final-Memo-Romanach.pdf. For privacy reasons, we also use a simulated landscape instead of using the true GPS coordinates of the sites.

There are five key habitats listed in Table 1. The target for each habitat is defined as a proportion of the available habitat in the initial landscape. We use the same proportion as observed for the EHNWR project for our simulated landscape (see Table 1).

**Table 1:** List of the 5 key habitats used for the comparison and proportion of the initial available habitat that should be reserved. The parameters of the Gaussian random field used to simulate the amount of habitat are also provided.

	Habitat	Target	Mean *m*^2^ (variance)	Range	Sill
*h*_1_	Dry prairies.	67%	51582 (153047)	0.888	0.954
*h*_2_	High pines, Florida scrub and sandhills.	38.8%	29836 (101547)	0.713	0.931
*h*_3_	Freshwater forested wetlands.	26%	71027 (143387)	0.58	1
*h*_4_	Mesic and hydric pine flatwoods and scrubby flatwoods.	32%	109553 (223443)	0.693	1
*h*_5_	Wet prairies and freshwater marshes.	38.8%	133791 (186932)	0.693	1
	Non-key habitat	0	604207 (351229)	0.793	0.986

To simulate habitat, we first compute the empirical variogram of each habitat based on their observed spatial distribution in central Florida. We then use this estimation to construct an exponential covariance matrix associated with each habitat. We simulate the value of a multivariate Gaussian vector, with mean and variance estimated from the true landscape and the covariance matrix computed from the estimated variogram. We normalize the amount of each habitat per site, such that the total amount of habitat does not exceed the site’s area. Finally, the boundaries of the sites are simulated using a Voronïo diagram. In the simulated landscape, “wet prairies and freshwater marshes” (*h*_5_) is the most frequent habitat while the four others are nearly present in the same proportion (see S1 Fig). Note that a large proportion of the first habitat (i.e. 67% of the initial amount) has to be reserved, thus habitat conversion can be critical to reserve the required amount of this habitat. Indeed, on average over the 1,000 simulated scenarios of habitat conversion, 37% of the first habitat is lost after only 8 years if not protected. In other words, there is not enough of the first habitat after 8 years to meet the habitat target if nothing was reserved before. More than 50% of the first habitat is lost after 13 years. The amounts of habitat are provided in S1 Fig. To determine the conversion rate, we first divide the sites into three different categories, depending on the amount of target habitats. The first category contains the sites with a cumulative proportion of target habitats lower than 30%. A cumulative proportion between 30% and 60% for the second category and between 60% and 100% for the third category. Then, for each category, we estimated from the data set the probability distribution over conversion rates. Finally, we drew the conversion rate of each site using the estimated probability distribution corresponding to the site’s category. The sites’ conversion rates are provided in S2a Fig. The data set does not exactly provide conversion rates, but urbanization forecasts to year 2060. This forecast provides projection of the future urban development due to sea level rise. We interpreted them as a measures of conversion rates as in [15]. For the sites’ costs, we fitted an exponential distribution to the observed site costs in the data set. The mean of the exponential distribution, which is the per km^2^ land cost, is estimated to US$25,242 (see S2b Fig). In this case, the cost of a site only depends on the site surface area and not on the amount of key habitats. We used the same stochastic budget as described in the previous experiment. We solved the DRDP with four different BLM values: BLM = 0, BLM = 500, BLM = 1,000 and BLM = 2,000.

The Matlab codes used to run all the experiments can be found in S2 File and the codes used to simulate landscape in S3 File.

## Results

### Comparison on a small landscape

Among the approximate solutions, the augmented greedy heuristics provide the best results (see Table 2). On average, they meet the habitat target with one or two fewer sites than the simple greedy heuristics in the non correlated and correlated scenarios.

**Table 2:** Expected extended cost for the comparison on a small landscape.

Strategy	Not correlated	Correlated
**Optimal**	4.76	6.78
Marxan	7.02	9.92
Greedy Rarity	6.09	9.53
Greedy Richness	6.1	9.53
Augmented Rarity	5.04	7.27
Augmented Richness	5.04	7.21

In the non-correlated scenario, conversion probability and amount of habitat are independent. In the correlated scenario, sites with high amount of the first habitat are more likely to have a high conversion probability.

**Fig 3:**
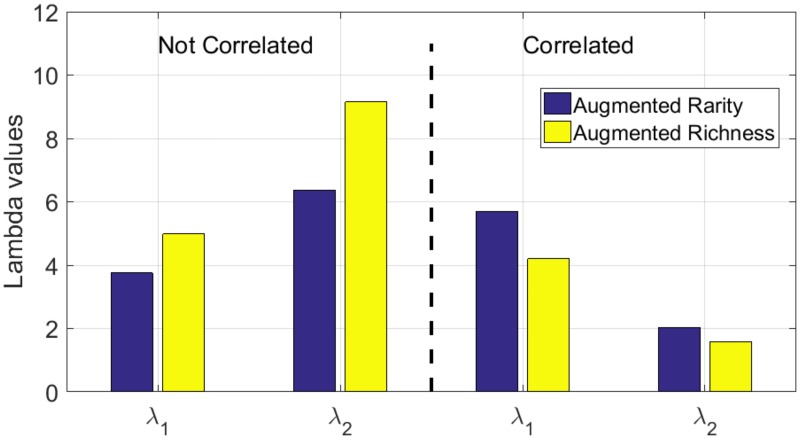
Value of the parameters λ_1_ and λ_2_ of the two augmented heuristics.

The value of the parameters λ_1_ and λ_2_ of the augmented heuristics are presented in Fig 3. Although the weights used by each strategy are different, both augmented heuristics give more weight to the second habitat in the non correlated scenario and they give more weight to the first habitat in the second scenario. In this case, the first habitat has a high conversion pressure and it thus allows prioritizing sites with more of the first habitat at the beginning, thus preventing the future effect of sites’ conversion.

The adaptive Marxan strategy has surprisingly low efficacy. This can be explained by the fact that when sites are selected, there is no consideration of the future possible conversion of land. This can unfortunately result in excluding valuable sites from the sequence.

For example, let’s consider a landscape with 4 sites, *s*_1_, *s*_2_, *s*_3_ and *s*_4_, all with unitary cost. Let’s consider only one key habitat with a target *G*_*H*_1__ = 7 and the following amount of habitat in each site: *h*_1_(*s*_1_) = 6, *h*_1_(*s*_2_) = 2, *h*_1_(*s*_3_) = 4 and *h*_1_(*s*_4_) = 3. Using Marxan, the networks ${\bar{N}}^{T}=\{s_{1},s_{3}\}$ and ${\bar{N}}^{T}=\{s_{3},s_{4}\}$ have an equal extended cost of 2 (i.e., the price of the network). There is thus no difference between these solutions in terms of value and Marxan can for example return the solution ${\bar{N}}_{\text{Marxan}}^{*}=\{s_{3},s_{4}\}$. Suppose that after computing the optimal ordering of the sequence, site *s*_3_ is purchased first. There are 8 possible different landscapes for the second time step, depending if sites *s*_1_, *s*_2_ and *s*_4_ are converted or not. On these 8 possibilities, there are 2 scenarios where it will not be possible to meet the habitat target: (i) if all sites are converted and (ii) if sites *s*_1_ and *s*_4_ are converted. But on the contrary, if the solution returned by Marxan was ${\bar{N}}_{\text{Marxan}}^{*}=\{s_{1},s_{3}\}$ and site *s*_1_ was purchased first, it would have been possible to meet the target in 7 cases out of 8. Note that a greedy heuristic would have selected site *s*_1_ first, which in this case would have increased the probability of meeting the habitat target.

### Comparison on a large landscape

#### Comparison without compactness

**Fig 4:**
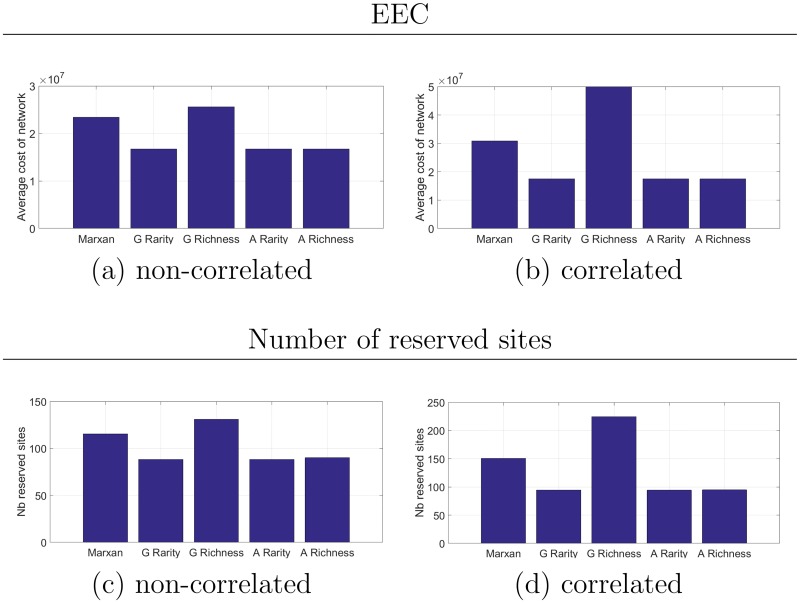
Results for the large landscape experiment without compactness. Expected extended costs of the reserved networks in (a) the non-correlated scenario and (b) correlated scenario. Average number of reserved sites in (c) the non-correlated scenario and (d) correlated scenario.

For both conversion scenarios, the greedy heuristic using the rarity criterion and the two augmented heuristics provide similar results and perform best among all the tested strategies (see Fig 4a and 4b).

In general the tested strategies reached the habitat targets except in the correlated scenario where the Marxan heuristic did not reach the target for the first habitat on 3 trajectories and on 33 trajectories for the greedy heuristic that uses the richness criterion. A particularity of the simulated landscape is that the first habitat is rare and the second habitat is present in a large quantity. When the selection of sites is based on richness, sites with a large amount of the second habitat tend to be selected first. Indeed, the amount of first habitat in sites is generally too low to influence the richness criterion. In this case, the best sites in terms of the first habitat are selected later and unfortunately some of the most valuable sites might have already been converted. Note that the same phenomenon is observed with the Marxan heuristic when the optimal order is also based on a richness criterion. Nonetheless, conversion probabilities are also accounted for, which certainly allows the policy to be more efficient. In all cases, both the greedy heuristic based on richness and the Marxan heuristics need to purchase more sites to reach the habitat targets (see Fig 4c and 4d). Even in the non-correlated scenario, this rule of selecting first the valuable sites in terms of the second habitat increases the number of sites needed to reach the targets, but the difference is greater in the correlated scenario.

For this landscape set-up the heuristic based on rarity is, unsurprisingly, one of the most efficient strategies, as long as the criterion accounts for the fact that the first habitat is rare. In this case, valuable sites for this habitat are selected first, thus preventing site conversion. The efficiency of the augmented heuristics are very similar to the greedy heuristic based on rarity. It is interesting to note that the augmented richness heuristic again uses λ_1_ > λ_2_ in the correlated scenario, such that valuable sites for the first habitat are selected first.

For the next comparison, only the results for the augmented rarity heuristic are computed, so as to save computation time, since the two augmented heuristics provide similar results.

#### Comparison with compactness

**Fig 5:**
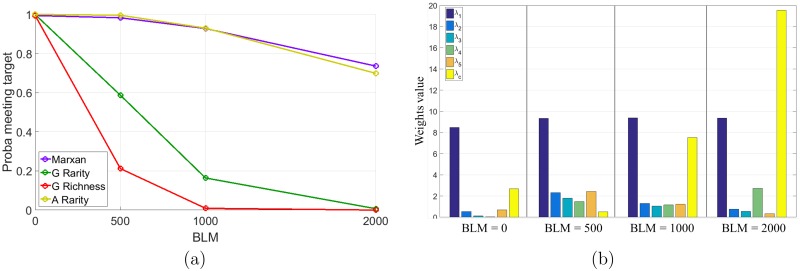
Results for the large landscape experiment with compactness. (a) Probability that the policy allows to meet the habitat targets. (b) Value of the weights used by the augmented rarity heuristic.

Increasing the BLM value forces the policies to construct reserve networks that are highly connected. A possible side effect is that the goal of meeting the habitat target for a minimal cost will become less important than having a compact network. When sites can be converted during the construction of the network, large BLM values can lead to situations where some of the habitat targets are not met before the habitat has been entirely converted (see Fig 5a). For BLM = 2,000 the greedy richness and rarity heuristics are highly affected and they do not meet the habitat targets in nearly any simulated scenarios. The Marxan and augmented rarity heuristics are less affected by the BLM value, even if the habitat targets were not met in nearly 30% of the simulated trajectories when BLM = 2,000. For the simulated landscape that we used, the first habitat target is the hardest to meet and it is the only target that is missing.

**Fig 6:**
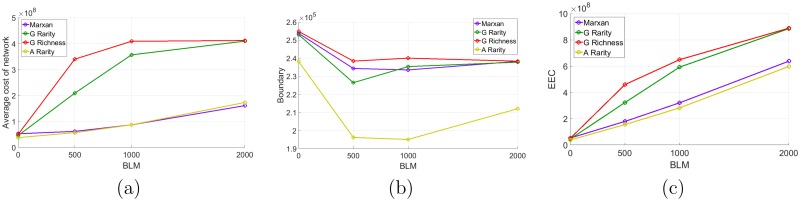
Results for the large landscape experiment with compactness. Cost of the reserved network (b) Value of the boundary computed on the reserved network (see Eq 11). (c) Estimated value of the expected extended cost (see Eq 4).

Among the greedy heuristics, again the one based on the rarity criterion is more efficient (see Fig 6).

But this time, the difference between the greedy and augmented rarity heuristics is much greater when BLM is high. The augmented rarity policy is the one providing the best results in terms of network compactness (see Fig 6b) and expected extended cost (see Fig 6c). But it is important to note that the efficiency of the Marxan policy and the augmented rarity are much closer in this case. In fact, the Marxan policy is even performing better in terms of $ cost when BLM = 2,000 (see Fig 6a). This is explained by the fact that the Marxan strategy is more likely to meet the habitat target in this case (see Fig 5a).

The augmented rarity is using a value of the weight λ_1_ which favors the first habitat, for which the habitat target is the hardest to meet (see Fig 5b). The value of λ_*C*_ is also increasing for BLM = 500 to 2,000, which reflects the increasing influence of having a network as compact as possible. One can see that the efficiency of the augmented rarity heuristic is surprisingly decreasing in terms of boundary value from BLM = 1,000 to BLM = 2,000. It is explained by the fact that for BLM = 2,000 meeting the habitat targets becomes less important in the process of selecting the reserved sites. As explained earlier, a side effect is that valuable sites in terms of the first habitat are lost before being reserved. In this case, all policies need more sites to meet the target (when possible), which indirectly increases the value of the boundary. For example, the augmented heuristics is purchasing on average 264 sites for BLM = 1,000 when it is 389 for BLM = 2,000. The marxan heuristics is purchasing on average 451 sites for BLM = 1,000 and 522 for BLM = 2,000; note that the augmented heuristics is purchasing far fewer sites, which explains the better efficacy in terms of compactness.

Finally, it is interesting to note that the five policies have quite different behaviors (see the sites’ selection frequency in S3 Fig. The greedy heuristics are highly influenced by habitat conversion and as a result, the reserved network can be very different between two land conversion scenarios. In contrast, the augmented rarity seems to be quite stable among the different simulated trajectories.

## Discussion

This article presents different solution methods for the problem of dynamic construction of a reserve network that minimizes cost while meeting habitat targets. Although commonly needed in practice, relatively few solutions are available for problems of large size. By nature the DRDP is computationally costly, and with current technology there is no hope that exact solution methods can be used, even for modest problems (e.g. one hundred sites). We proposed different heuristics that can be run for large problems. We first proposed a natural extension of the Marxan solution that accounts for the conversion rate of the selected sites. To do this, we first computed the optimal network with Marxan at each decision step, and then computed the optimal purchasing order using the principle of the site-ordering algorithm [24]. In addition, we proposed an augmented version of two common naive myopic heuristics. At each decision step, these heuristics define the value of each site *V*(*s*) as a linear combination of *features* $V\left(s\right)=\sum_{j=1}^{J}f_{j}\left(s,{\bar{N}}^{t},S^{t}\right)$, where each feature *f*_*j*_ is based on a richness or rarity criterion. In some problems, some biodiversity features should be selected first, either because of high conversion rates and/or because of habitats rarities, which we proposed to model using weights λ_1_, …, λ_*J*_ in front of each feature function: $V^{\prime }\left(s\right)=\sum_{j=1}^{J}\lambda_{j}f_{j}\left(s,{\bar{N}}^{t},S^{t}\right)$. To compute the weights, we proposed to combine a simulation approach with a genetic algorithm, such that each biodiversity feature weight λ_*j*_ is automatically fitted according to the specificity of the decision problem.

In order to test this method and verify that weights were actually learned from the structure of the problem, we designed two simulated examples (small and large landscapes with BLM = 0), where one habitat was rare and subject to a high conversion probability and showed that the weights for this habitat were higher, allowing the augmented heuristics to be the best tested strategies. One drawback of the naive myopic strategies is that cost, biodiversity and compactness measures are all mixed in one same criterion. Thus, we also proposed to use weights for the cost and boundary length criterion to automatically scale them with the biodiversity features. We showed on the last experiment with BLM > 0)that this drastically increases the performance over the non-augmented greedy strategies and even outperformed the dynamic version of Marxan.

One general conclusion of this work is that the augmented greedy strategies are able to automatically learn from the structure of the problem to weight biodiversity, cost and compactness measures in order to outperform the common greedy strategies. However, when compactness is not accounted for and conversion probabilities are approximately equal among the biodiversity features, the augmented and non-augmented greedy heuristics are likely to provide similar results. On the example inspired from the extension of the EHNWR, the augmented greedy heuristic and dynamic Marxan exhibit similar performances, although the augmented heuristic allows much better performance in terms of boundary length and thus compactness. These encouraging results suggest that the augmented heuristics can provide useful reserve design policy to decision makers.

Other solutions might also be investigated. For example, the principle that we used to extend the Marxan solution to a dynamic problem can also be used to extend the solution computed with integer linear programming. Indeed, the static reserve design problem can also be formulated as an Integer Linear Programming (ILP) problem (see for example [10]) to compute a solution of the static problem. Then, ${\bar{N}}_{\text{Marxan}}$ can be replaced with ${\bar{N}}_{\text{ILP}}$, the solution provided by any software suitable for solving ILP problems. Criteria other than rarity or richness can also be used, such as the irreplaceability criterion [22].

When compactness is accounted for, defining an adapted BLM value seems to be critical. In our last experiment, we showed that with the augmented rarity heuristic, BLM = 500 greatly improves the compactness of the network without significantly increasing the cost (in $). On the other hand, for BLM = 1,000 the gain in compactness appears to be small while the network cost (in $) is nearly double compared to the case where BLM = 0. Finally, for BLM = 2,000 the efficiency in terms of compactness is reduced while the network cost is significantly increased, showing that neither the compactness nor the price increase linearly with the BLM. In practice, applying different policies on simulated land conversion trajectories might be a useful way to determine the best BLM value that represents the best compromise between compactness and cost of the network. Also note that we used the boundary length as a criterion to quantify the compactness of a network, in order to be able to compare with the Marxan solution. But this might not be suitable in some situations, as for example marine protected areas [32], but different criterion for compactness or connectivity can be used to define the augmented heuristics [33, 34], given they are relatively easy to compute as far as our method relies on intensive simulations.

## Supporting information

S1 Fig Spatial distribution of the five targets habitats in the large landscape with compactness example. The gray color indicates the proportion of habitat in the site, such that a white (black) site indicates that the habitat is not present in (fully cover) the site.
(EPS)

S2 Fig (a) Conversion rates in the large landscape with compactness example and (b) cost of the sites. For (a), the gray color indicates the conversion probability, from white (*μ* = 0) to black (*μ* = 1). For (b), the cheapest site is in white, while the most expensive one is in black.
(EPS)

S3 Fig Site selection frequency in the large landscape with compactness experiment with BLM = 500. The frequency is represented in gray scale, meaning that black sites are always selected for the 1,000 simulated trajectories of land conversion, while white sites are never selected by the policy.
(EPS)

S1 File Comparison_Costello_Polasky.pdf. Details and results of the comparison with the Costello & Polasky problem [15].
(PDF)

S2 File ComparisonPresentedInTheArticle.zip. All the necessary Matlab codes to run all the experiments presented in the article.
(ZIP)

S3 File LandscapeSimulation.zip. All the necessary Matlab codes to simulate landscape.
(ZIP)
