# Supplementary material for: Dynamic minimum set problem for reserve design: Heuristic solutions for large problems
Source: PLoS One. 2018 Mar 15;13(3):e0193093. doi: 10.1371/journal.pone.0193093 (PMC5854297; doi:10.1371/journal.pone.0193093)
Supplement: S1 File — Details and results of the comparison with the Costello & Polasky problem [15]. (PDF) [file pone.0193093.s004.pdf]

Comparison with the Costello and Polasky problem

. We applied the augmented and non-augmented richness strategies to the problem defined in [1], section 4. For this problem, $h_j(s)$ represents the presence of species j in site s and $H_j = 1,\ \forall j = 1,\dots,28$. In this case, the reward function of the richness heuristic is:

$$r^t_{Rich_j}(s) = \begin{cases} 1 & \text{if species } j \text{ is present in site } s \text{ and not present in} \\ & \text{the reserve network.} \\ 0 & \text{otherwise} \end{cases}$$

For this problem, there is: only 7 available sites, a unitary site's costs and budget, a fixed planning period of 4 years. The conversion rates are randomly drawn from a uniform distribution $U([0.2, 0.7])$ and the occurrence of species is determined as follows:

$$h_j(s) = \begin{cases} 0 & \text{if } z_{js} > 0.3. \\ 1 & \text{if } z_{js} \leq 0.3. \end{cases}$$

Where z_{js} is a realization of a uniform distribution $U([0,1])$.

The value of a reserve design policy is equal to the expected number of species present in the landscape at the beginning of year $T+1$, where $T = 4$ is the planning period. Thus, we computed the number of species still present in the network, only after the first four years of sites selection and development. As in [1], the value of the augmented and non-augmented heuristics are computed over 100 simulated landscapes (i.e. 100 simulated values of $(\mu(s), h(s))\ ,\ \forall s = 1\dots7)$, but for each landscape we ran 100 scenarios of sites conversion, such that the value of each policy is estimated over 1000 strategies instead of only 100. In [1], the optimal strategy has a value of 20.65 (S.D. 1.73) and the naive myopic has a value of 20.19 (S.D. 1.79), in other words, the optimal strategies allows an increase of 2.2% compared to the naive myopic. In our experiment, the augmented richness strategy has a value of 20.194 (S.D. 1.81) and the naive myopic of 20.008 (1.8), in other words the augmented richness strategy allows an increase of 0.9% over the naive myopic. It is hazardous to compare the values from the original article to our values as far as they were not computed on the same data set. But given that there is 2.2% difference between the optimal and the naive myopic and 0.9% difference between the augmented richness and myopic heuristic, one possible interpretation is that the augmented richness strategy is approximately half-way between the optimal and the naive myopic.

It is difficult to analyze raw values of the weights used by the augmented heuristic strategy but we tried to show that this strategy automatically adapt choices, such that richness is not the only decision criterion. For all simulated trajectories, we stored the purchasing year of the reserved sites as well as their ranks, in terms of richness and conversion probability. First, we computed Kendall's tau coefficient [2], in order to quantify the correlation between purchasing year and site's richness. Note that although the coefficient should intuitively be equal to 1 for the naive myopic, it is not the case because sites with initial low richness rank can be selected when sites with initial high richness rank are converted, and thus not available for the reserve The coefficient is equal to $\tau_{NM} = 0.67439$ for the naive myopic strategy and to $\tau_{AG} = 0.52848$ for the augmented richness strategy. One can see that $\tau_{NM} > \tau_{AG}$, which tends to show that richness ranks is not the only site selection criterion for the augmented richness strategy. Second, we fitted a linear model expressing the purchasing year as a linear combination of the richness rank and the conversion probability rank. The fitted coefficient are $(r_{NM}, c_{NM}) = (0.51752, 0.16899)$ for the richness rank and conversion probability in the case of the naive myopic strategy and $(r_{AG}, c_{AG}) = (0.31561, 0.24939)$ in the case of the

augmented richness strategy. Again, $r_{NM} > r_{AG}$, meaning the richness rank tends to be more important for the naive myopic. On the contrary, $c_{NM} < c_{AG}$, such that the augmented greedy strategy is more influenced by the conversion probability and is easily selecting sites with lower richness but with higher conversion probability. To conclude, the augmented greedy heuristic is able to automatically enhance the naive myopic, in a direction that allows increasing the expected strategy value.

References

1. Costello C, Polasky S. Dynamic reserve site selection. Resource and Energy Economics. 2004;26:157–174.

2. Kendall MG. A new measure of rank correlation. Biometrika. 1938;30(1/2):81–93.
