# Supplementary material for: Dynamic minimum set problem for reserve design: Heuristic solutions for large problems
Source: PLoS One. 2018 Mar 15;13(3):e0193093. doi: 10.1371/journal.pone.0193093 (PMC5854297; doi:10.1371/journal.pone.0193093)
Supplement: S2 File — All the necessary Matlab codes to run all the experiments presented in the article. (ZIP) [file pone.0193093.s005.zip › ComparisonPresentedInTheArticle/ComparisonWithCompactness/README.docx]

In this folder are all the functions allowing to run the example on a large landscape with connectivity.

The optimal set of weights of the two augmented heuristics should be computed first using ComputeOptimalWeights.m

This function uses ValueLambda_Rarity.m and ValueLambda_Richness.m to approximate the value of a strategy for a given set of weights.

Then, main_EstimateValue.m should be used to estimate the value of the augmented Rarity and Richness as well as the two associated greedy strategies.

The .mat files are the landscapes setup.
