# Supplementary material for: Dynamic minimum set problem for reserve design: Heuristic solutions for large problems
Source: PLoS One. 2018 Mar 15;13(3):e0193093. doi: 10.1371/journal.pone.0193093 (PMC5854297; doi:10.1371/journal.pone.0193093)
Supplement: S2 File — All the necessary Matlab codes to run all the experiments presented in the article. (ZIP) [file pone.0193093.s005.zip › ComparisonPresentedInTheArticle/ComparisonWithPolasky/README.docx]

main_Polasky should be run to estimate the value of the augmented heuristics computed on the problem presented in [1], section 4.

ValueLambda_Polasky.m is the function that estimate the value of a strategy, given a set of weights

[1] COSTELLO, Christopher et POLASKY, Stephen. Dynamic reserve site selection. *Resource and Energy Economics*, 2004, vol. 26, no 2, p. 157-174.
