# Supplementary material for: Dynamic minimum set problem for reserve design: Heuristic solutions for large problems
Source: PLoS One. 2018 Mar 15;13(3):e0193093. doi: 10.1371/journal.pone.0193093 (PMC5854297; doi:10.1371/journal.pone.0193093)
Supplement: S3 File — All the necessary Matlab codes to simulate landscape. (ZIP) [file pone.0193093.s006.zip › LandscapeSimulation/README.docx]

SimulationOfLandscape.m is the main script allowing to simulate a landscape as well as to simulate trajectory of budget and site conversion.

randomfield.m and correlation_fun.m are two functions used to simulate a Gaussian random field. These functions were created by Paul Constantine and were downloaded on the [MathWorks File Exchange website](https://fr.mathworks.com/matlabcentral/fileexchange/27613-random-field-simulation?focused=5229524&tab=function).
